# Morphologic perfusion patterns and PI-RADSv2.1 in transition zone prostate cancer

**DOI:** 10.1007/s00261-023-04021-w

**Published:** 2023-08-28

**Authors:** M. Garmer, D. Grönemeyer, Th. van de Loo, S. Mateiescu, D. Schaffrin-Nabe, P. Haage, L. Kamper

**Affiliations:** 1 Radiology Private Practice, Universitätsstr. 110E, 44799 Bochum, Germany; 2 Radiology Department, St. Elisabeth Hospital Herten, Herten, Germany; 3 Grönemeyer Institute of Microtherapy, Universitätsstr. 142, 44799 Bochum, Germany; 4 Oncology Private Practice, Universitätsstr. 110E, 44799 Bochum, Germany; 5 Radiology, Helios University Hospital Wuppertal, Wuppertal, Germany; 6 Witten/Herdecke University, Witten, Germany

**Keywords:** Prostate cancer, Diagnostic imaging, Multiparametric MRI, Perfusion-weighted MRI, MRI, Interventional, Image-guided biopsy

## Abstract

**Purpose:**

To evaluate morphologic perfusion patterns in transition zone prostate cancer in multiparametric MRI controlled by in-bore MRI-guided prostate biopsy.

**Methods:**

Two experienced radiologists evaluated MRI perfusion patterns in consensus from 321 biopsy cores from the transition zone in 141 patients. Transition zone cancer was present in 77 cores in 36 patients. Single early-phase perfusion images were evaluated separately for the presence of a transition zone prostate cancer (consensus tumor early perfusion). The proposed criteria for the perfusion pattern (asymmetry, signal strength, and homogeneity) were rated in consensus for each biopsy position in the presence of the T2w images including the markers of the biopsy trace. We analyzed receiver operating characteristic curves for the PI-RADSv2.1 score and the proposed perfusion pattern.

**Results:**

A logistic regression model with PI-RADSv2.1 and perfusion patterns in early perfusion imaging improved the model fit significantly compared to a model containing only PI-RADSv2.1 (Likelihood Ratio Test, LR = 14.5, *p* < .001). The AUC was 0.96 for the multiple regression model compared to 0.92 for the PI-RADSv2.1 alone. The evaluation of homogeneity in single early-enhancement images is not inferior compared to the conventional DCE parameter of PI-RADSv2.1 (AUC 0.84 versus 0.83).

**Conclusion:**

Morphologic perfusion patterns significantly improve the diagnostic performance of PI-RADSv2.1 in TZ prostate cancer.

**Graphical abstract:**

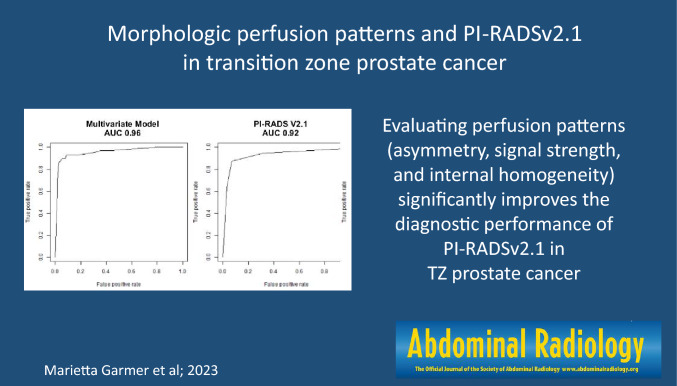

## Introduction

Prostate cancer still remains the second most diagnosed cancer among males worldwide [1].

Multiparametric prostate MRI has gained a leading role in the diagnosis of prostate cancer. The decision whether to perform a prostate biopsy or not is increasingly based on MRI results besides clinical factors. Targeted biopsies according to MRI results are more and more included in systematic biopsies [2]. Standards for the MRI protocol and structured reporting have evolved (PI-RADS–Prostate Imaging Reporting and Data System) over years. The PI-RADS is considered to be continuously optimized by experience and objective data [3].To date, version 2.1 (PI-RADSv2.1) has emphasized the value of T2-weighted (T2w) imaging for the transition zone and diffusion-weighted imaging (DWI) for the peripheral zone [4].

Transition zone (TZ) prostate cancer is much less frequent (less than 30% of all prostate cancer) than peripheral zone prostate cancer. Due to the anatomic position, it is difficult to diagnose TZ prostate cancer by a digital rectal exam, and multiparametric MRI is challenging in the TZ [5–7]. Benign prostatic hyperplasia (BPH) is characterized by the formation of nodules in the transition zone. Different histologic subtypes of glandular and fibromuscular/stromal proliferation lead to a heterogeneous appearance in T2w images [8]. For the TZ, there is an elaborated description of T2w patterns in PI-RADSv2.1. Suspicious lesions have a lenticular shape or a structure like “erased charcoal,” meaning loss of internal heterogeneity. Atypical nodules with predominant stromal hyperplasia may be misdiagnosed as suspicious lesions [4].

The value of dynamic contrast-enhanced DCE imaging is limited in this context according to PI-RADSv2.1.

DCE analysis by signal–time curves and pharmacokinetic models were described in PI-RADSv1 [9]. Because of missing evidence, this classification is no longer recommended for the clinical use. The assessment of DCE in PI-RADSv2.1 and PI-RADSv2 yields a distinction between positive and negative scores. A positive score means perfusion, that is, “focal, and; earlier than or contemporaneously with enhancement of adjacent normal prostate tissue; and; corresponds to suspicious finding onT2w and/or DWI. ” This positive DCE score is only effective in the PZ and does not play a role in the scoring of the TZ. However, there are empirical data that DCE may also have a role in the TZ [4]. Evaluation of DCE positivity for T2w imaging in the transition zone has already shown higher cancer detection rates [10, 11].

The Gleason grade is a well-known grading system that describes the histologic pattern of cellular arrangement in prostate cancer. T2w patterns in PI-RADSv2.1 and the Gleason grade refer to discrepancies to a normal glandular pattern. The most important histologic features of prostate cancer that may show a correlation to MRI signs, are neoangiogenesis and increased cellular density. Neoangiogenesis leads to highly irregular neovessels with increased permeability. Higher perfusion of cancer lesions can be described on this basis by early enhancement followed by washout. Increased cellular density means reduced extracellular space and reduced luminal volume [8]. It could be assumed that these changes in the glandular pattern also lead to further specific changes in the perfusion pattern with a view to heterogeneity or homogeneity. Perfusion patterns could be interesting for texture analysis as shown by Sun et al. for T2w imaging [12].

**Fig. 1:**
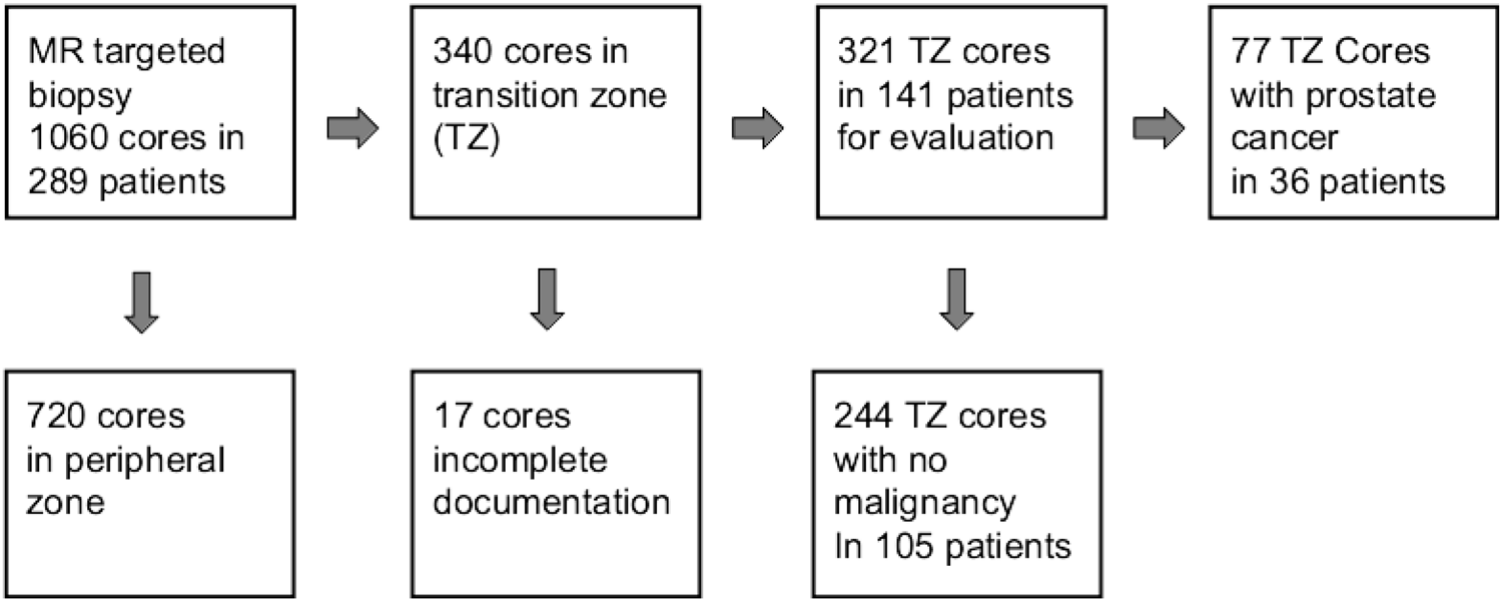
Flowchart of patient inclusion and exclusion

Malignant lesions are supposed to be asymmetric, whereas BPH represents diffuse symmetric changes in the prostate. The early enhancement of cancer lesions leads to a higher signal strength than the surrounding benign tissue in early-phase perfusion images. A benign pattern can be described as a diffuse pattern with a symmetric collection of rings and arcs or bubbles, with some blurry nodules, and an appearance like cotton-wool, or “popcorn” as proposed by Rosenkrantz et al. (Fig 1). Blurry nodules in a benign glandular pattern mean smooth borders. A malignant pattern is often focal with internal homogeneity or “sheetlike” as proposed by Rosenkrantz et al. [11]. This can best be described as a loss of the benign glandular pattern with a loss of rings, arcs, and bubbles opposed to a more or less homogenous enhancement with a blurry internal aspect and sharp borders. We summarize this appearance as internal homogeneity. This feature of internal homogeneity together with asymmetry and signal intensity gives a more complex description of the visual perfusion assessment.

We propose characteristic perfusion patterns for TZ cancer and performed a consensus evaluation for asymmetry, signal strength, and internal homogeneity in a dataset controlled by in-bore MRI-guided biopsy.

## Methods and materials

The local ethics committee approved this retrospective study. Written informed consent was obtained from all patients.

### Study design

From 2007 until 2020, in-bore MRI-guided prostate biopsy was performed in 289 consecutive patients with suspected prostate cancer.

According to the MR findings, we obtained 3 to 6 cores per patient. From the resulting 1060 cores, 340 cores from the TZ could be identified. A total of 17 cores had to be excluded because of incomplete documentation.

321 cores in 141 patients remained for evaluation (Fig.1).

Two experienced radiologists reviewed all image sets in in a blinded consensus. One of both radiologists with more than 15 years of experience in prostate imaging performed all biopsies since 2008.

**Fig. 2:**
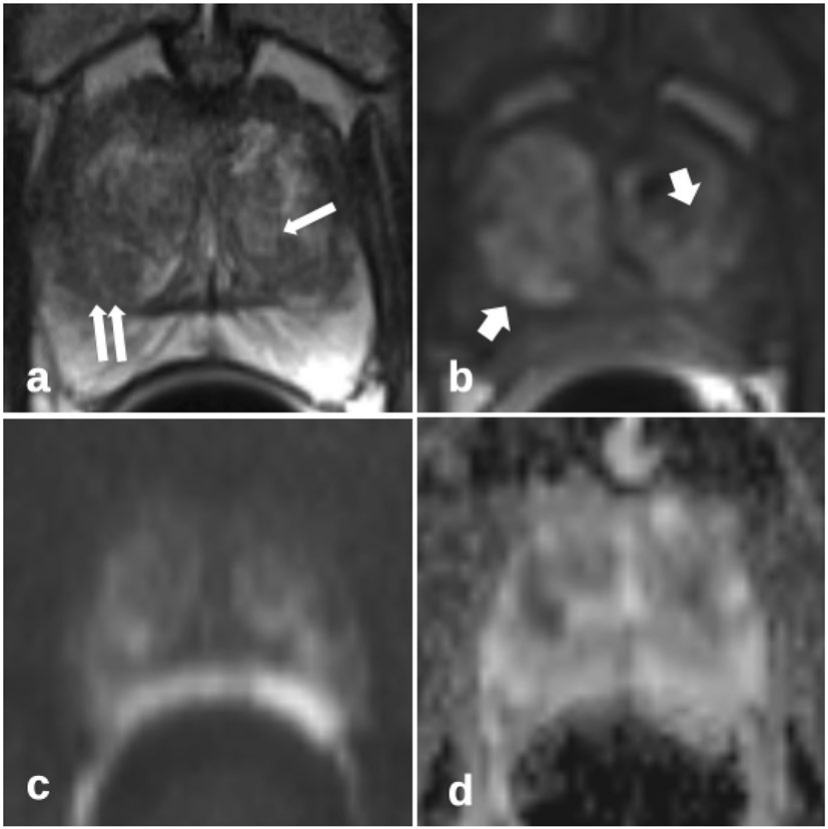
Unspecific heterogeneous perfusion pattern (short arrows in **b**) of the transition zone in benign prostate hyperplasia, without marked asymmetry and marked high signal intensity; patient of 57 years, PSA 5.1 ng/ml, prostate volume 57 ml, no carcinoma; **a** T2-weighted imaging axial with marked biopsy traces (long arrows); **b** T1-weighted early-phase dynamic perfusion; **c** diffusion-weighted imaging with b = 1400; **d** apparent diffusion coefficient map

**Fig. 3:**
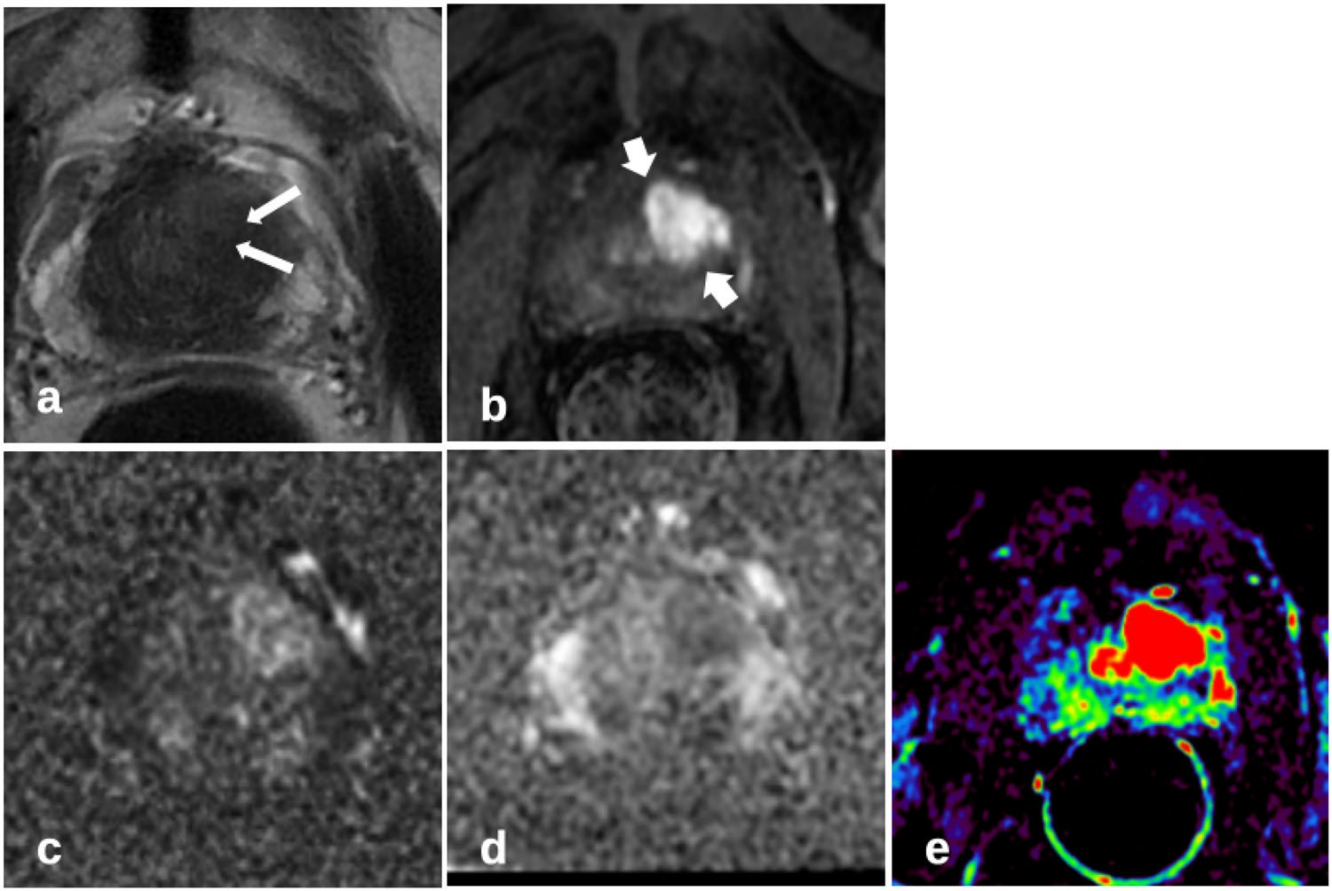
Typical tumor perfusion pattern (short arrows in **b**) of the left anterior transition zone, with marked asymmetry and with marked high signal intensity, moderate reduced heterogeneity; patient of 70 years, PSA 39 ng/ml, prostate volume 36 ml, prostate carcinoma G2 Gleason 3+4; **a** T2-weighted axial imaging with marked biopsy traces (long arrows); **b** T1-weighted early-phase dynamic perfusion; **c** diffusion-weighted imaging with b = 1400; **d** apparent diffusion coefficient map; **e**: calculated dynamic enhanced integral EI colour map

Single early-phase perfusion images were evaluated separately for the presence of a TZ prostate cancer (consensus tumor early perfusion). This was based on a combination of the image criteria for the whole slice. Afterward, the T2w images including the markers of the biopsy trace were presented to describe the perfusion pattern for each biopsy position in detail. The criteria for the perfusion pattern were (1) asymmetry; (2) signal strength compared to the surrounding tissue of the TZ and (3) homogeneity versus internal heterogeneity (Figs. 2 and 3).

Furthermore, the calculated dynamic color maps were evaluated for the presence of a TZ prostate cancer to take into account the dynamic character of the perfusion sequence (consensus tumor color map).

### Patient demographics and clinical data

Patients with suspicious lesions underwent biopsy after a consensus-based decision by a radiologist and a urologist on an individual base. The main criteria were the level and time course of PSA, together with the image interpretation of mpMRI, which was related to the respective guidelines/PI-RADS version. All imaging and interventional procedures were performed according to current guidelines. Further systematic biopsies before or after the targeted biopsy were not subject of this study. We targeted at least one lesion with the highest suspicion on the left and the right sides of the prostate.

The automatic biopsy gun provides a notch length of 17 mm. Thus, the position of the targets was retrospectively documented in up to 4 subsequent T2w slices of the preceding diagnostic MRI (long arrows in Figs.1a and 2a). All cores were retrospectively reevaluated according to PI-RADSv2.1. For this study, we evaluated exclusively cores of the TZ.

#### Sample description

A sample of 321 biopsy cores from 141 patients, 36 of which were carcinoma positive, i.e., had at least one core with neoplastic alterations, was analyzed. Overall, 77 cores were positive for prostate carcinoma. Tumor grading was G1, G2, and G3 in 14, 42, and 20 cores, and Gleason score was 3 + 3, 4 + 3, 3 + 4, 4 + 4, 5 + 5 in 32, 21, 16, 6, and 1 cores; for one core, no Gleason score was provided. Descriptive characteristics are summarized in Table 1.

**Table 1:** Descriptive characteristics of the carcinoma positive and negative patient subgroups

	Negative patients (*n *= 105)	Positive patients (*n* = 36)	*p*-value
Age [years]	64.8 ± 8.2, [39.0, 83.0]	69.5 ± 6.3, [58.0, 87.0]	0.02*
Number of cores	2.1 ± 1.2, [1.0, 6.0]	2.8 ± 0.9, [1.0, 5.0]	< 0.001*
Positive cores [%]	0.0	69.3	< 0.001*
Biopsy preceding [% yes]	29.7	48.5	< 0.001**
Biopsy preceding [% NA]	42.2	3	< 0.001**
PSA value [ng/ml]	9.3 ± 6.6, [1.5, 31.9]	15.1 ± 10.5, [4.5, 39.0]	0.007*
Prostate volume [ml]	53.7 ± 19.1, [20.0, 102.0]	54.4 ± 34.1, [23.0, 175.0]	0.242*

For continuous variables, the mean ± standard deviation, [min, max] is given. For categorical variables, the percentages of each category are given.

*Mann–Whitney-*U*-test,**chi-squared test

### MpMRI of the prostate and in-bore MR-guided biopsy

MR imaging was performed using a 1.5 Tesla (ESPREE, Siemens Healthineers) in 119 patients with endorectal coil and a 3-Tesla (PIONEER, GE Healthcare) scanner in 22 patients.

The standard protocol fulfilling the requirements of the Consensus Meeting on the Standardization of Prostate MRI and the appropriate version of the prostate imaging included T2w imaging in three planes. DWI was performed using four b-values (0, 100, 800, 1400 in 1.5 T and 0, 100, 800, 1500 in 3 T). The apparent diffusion coefficient was calculated from b = 0, 100, and 800 s/mm2. Volume-interpolated gradient echo sequences with a temporal resolution of 8.3 s were used for DCE in 1.5T. The 3 T scanner used a 3D two-point Dixon (DynamIc SCan Optimization DISCO) with a temporal resolution of 8.3 s.

Gadoteric acid was applied as contrast media in a weight-adapted standard dose (0.1 mmol/kg body weight) with an injection rate of 3 ml s^−1^. The protocol parameters of the DCE sequences are listed in Table 2.

**Table 2:** Protocol parameters of the DCE sequences

Field strength	Sequence	Plane	TR/TEFA	Slice Thickness	Matrix	FOV	Temporal resolution
1.5	VIBE	Axial	4.9/1.7/15 °	3 mm	192 x 144	260 x 260	8.3 sec
3	DISCO	Axial	6.5/1.7/15 °	3 mm	192 x 192	260 x 260	8.3 sec

Post-processing of all DCE datasets was carried out on a GE Advantage Workstation HPZ 800. For a representation of the perfusion kinetics in a fast visual assessment, we created simple colour maps as maximum slope images MSI and enhanced integral EI maps with constant settings.

For the biopsy, the patient was positioned using a biopsy positioning device in the supine position in the 1.5 Tesla scanner (preproduction model by Invivo) or in the prone position in the 3-Tesla scanner (DynaTRIM by Philips).

An adjustable needle guide was inserted transrectally under the control of sagittal and coronal fast imaging. In the correct position, the biopsy was performed using an 18-G MR-compatible fully automatic biopsy gun.

Each biopsy procedure was documented by fast T2w imaging in axial plane, and sagittal or coronal plane with the needle inside the lesion. All biopsy specimens were evaluated in the same histopathologic institute including Gleason grading of prostatic carcinoma.

### Data analysis

#### Statistical analysis

We used logistic regression to predict the histological status (cancer vs. no cancer) for each core. As (bivariate) predictors, we used the PI-RADSv2.1 score, its three constituents (V2.1 score T2w, V2.1 score DWI, V2.1 score DCE), lesion size (only available for carcinoma positive patients) as well as the diagnostic consensus variables (positive or negative) based on asymmetry, signal strength, homogeneity, early perfusion imaging, colormap imaging, MSI, and EI, respectively. Using the PI-RADS score and the diagnostic consensus variables as predictors, we subsequently fitted a multiple logistic regression model. From each model’s predictions, we calculated sensitivity, specificity, and receiver operating characteristic (ROC) curves, which graphically represent the different trade-offs between sensitivity and specificity that can be realized by a given predictive model, and their respective areas under the curve (AUC), which vary between 0.5 (chance level predictions) and 1 (perfect predictive accuracy of the model).

## Results

**Fig. 4:**
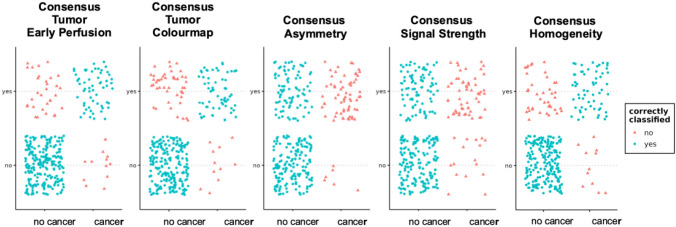
Scatterplots showing the distribution of the consensus results of early perfusion images and color maps including the single perfusion pattern characteristics asymmetry, signal strength, and homogeneity about the cores’ histological result

**Fig. 5:**
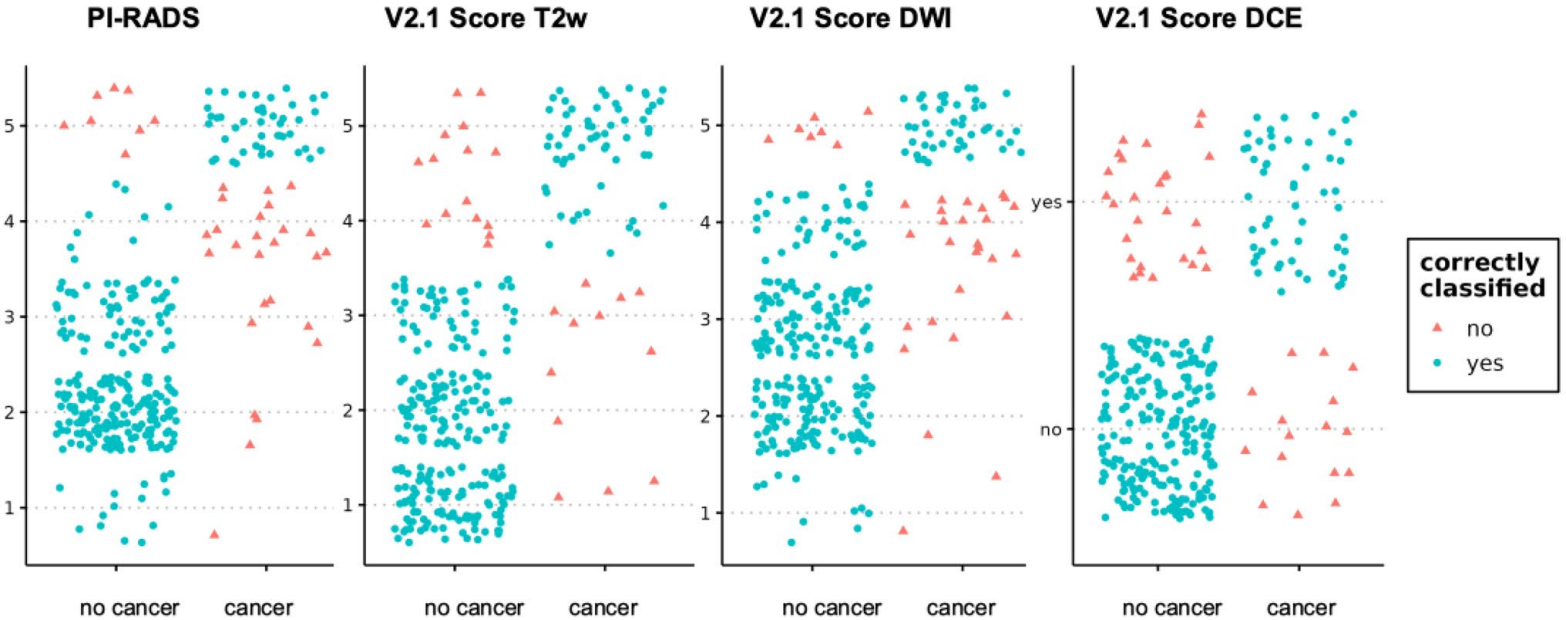
Scatterplots showing the distribution of the PI-RADSv2.1 score including the three constituents T2-weighted imaging, diffusion-weighted imaging, and dynamic contrast-enhanced imaging about the cores’ histological result

Figure 4 shows the distribution of the consensus results of early perfusion images and color maps about the cores’ histological status. Figure 5 shows the distribution of the results of the PI-RADS score and its three constituents.

Table 3 summarizes the results of the logistic regression models for each predictor variable.

**Table 3:** Summary of logistic regression models

	*n*	Significance	Sensitivity	Specificity	AUC
PI-RADSv2.1	321	*p* < .001	0.63	0.97	0.92
V2.1 score T2w	321	*p* < .001	0.83	0.94	0.92
V2.1 score DWI	321	*p* < .001	0.62	0.97	0.90
V2.1 score DCE	321	*p* < .001	0.77	0.89	0.83
Consensus asymmetry	321	*p* < .001	0*	1*	0.78
Consensus signal strength	321	*p* < .001	0*	1*	0.71
Consensus homogeneity	321	*p* < .001	0.85	0.84	0.84
Consensus tumor (early perfusion)	321	*p* < .001	0.83	0.88	0.86
Consensus tumor (colourmap)	321	*p* < .001	0.85	0.81	0.83
Consensus tumor (MSI)	321	*p* < .001	0.73	0.85	0.79
Consensus tumor (EI)	318	*p* < .001	0.81	0.82	0.81
Lesion size	100	*p* = 0.118	0.57	0.67	0.58
Multiple regression	318		0.87	0.96	0.96

For each bivariate predictor and the multiple logistic regression model, the number of available cores (*n*), the significance of the respective predictor within the logistic regression model, sensitivity, specificity, and AUC calculated from the logistic regression models’ predictions are given

*For consensus asymmetry and consensus signal strength, the best overall predictive accuracy was achieved when the model ignored those predictors and predicted the more frequent category (negative) for all cores. If the consensus variables were used despite leading to suboptimal predictive accuracy, a sensitivity/specificity of .92/.64 was achieved for consensus asymmetry, and of .77/.65 for consensus signal strength

The overall best bivariate predictor was V2.1 score T2w (AUC 0.92). The best specificity was achieved by V2.1 score DWI (0.97). The best sensitivity is given for the consensus homogeneity and the consensus tumor diagnosis based on colormap imaging (both 0.85). The multiple regression model achieved the overall best predictive accuracy (AUC 0.96). Thus, a combination of the variables improved the predictive accuracy. In particular, a model containing only PI-RADSv2.1 was significantly improved, when PI-RADSv2.1 was combined with an evaluation of early perfusion imaging for the presence of a TZ cancer (Consensus Tumor Early Perfusion) with a Likelihood Ratio Test, LR = 14.5 (*p* < .001).

While all predictors except lesion size were significant, their predictive value differed substantially. The footnote in Table 3 refers to the low predictive accuracy of the two predictors Consensus Signal Strength and Consensus Asymmetry caused by the unbalanced groups. This is also the reason for the scatterplots of these two features, where the logistic regression model simply classifies all patients as negative.

**Fig. 6:**
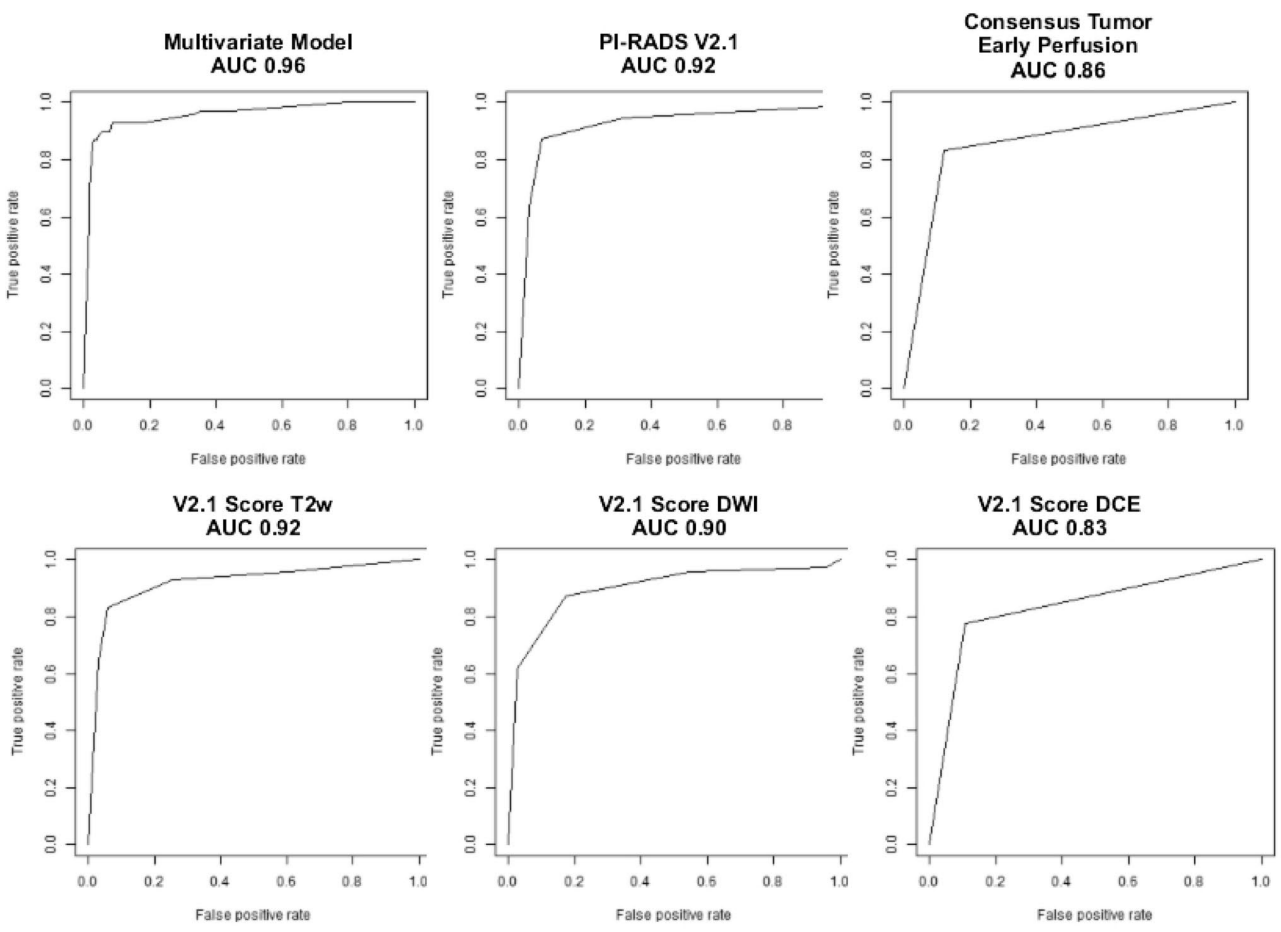
ROC curves for the multivariate model, PI-RADSv2.1, Consensus Tumor Early Perfusion, V2.1 score T2w, V2.1 score DWI, V2.1 score DCE; *ROC* receiver operating characteristic; *AUC* area under the curve; *T2w* T2-weighted; *DWI* diffusion-weighted, *DCE* dynamic contrast enhanced

Figure 6 shows ROC curves for some single models.

## Discussion

DCE characteristics are not part of TZ cancer classification in PI-RADSv2.1, though empirical data support a value of DCE in TZ cancer diagnostics [4].

In our study, we found a significantly improved diagnostic accuracy in the detection of TZ cancer when an evaluation of perfusion patterns was added to the PI-RADSv2.1 score (AUC 0.96 versus 0.92, *p* < 0.001).

Compared to our results, Wei et al. showed a lower AUC for the use of biparametric PI-RADSv2.1 without DCE of 0.866 for TZ cancer and 0.929 for clinically significant TZ cancer.

Greer et al showed improvement in cancer detection rates by adding DCE positivity to T2w imaging in transition zone cancers [10]. However, DCE positivity was limited to curve-type characteristics of the PI-RADSv2 definition, and the results were less relevant compared to the peripheral zone.

In contrast, Hoeks et al. did not show additional information on DCE in TZ cancer, even when parametric maps of pharmacokinetic modeling according to the model of Tofts were included [7].

In a recent study, Ziayee et al. evaluated qualitative, semi-quantitative, and quantitative analyses of DCE for cancer detection and differentiation of clinically significant cancer from non-significant cancer. Qualitative DCE according to PI-RADSv2.1 could distinguish cancer from lesions in the TZ, whereas semi-quantitative and quantitative analysis failed. The authors describe an AUC of 0.95 for PI-RADSv2.1 score and 0.92 for DCE according to PI-RADSv2.1. DCE according to PI-RADSv2.1 is related to simultaneous suspicious findings in T2w and/or DWI, which means, that these parameters are already included in DCE in their study. Clinically significant TZ cancer could not be distinguished [13], whereas Mirak et al. described a possible utility of qualitative and quantitative DCE for the detection of significant TZ prostate cancer [14].

The first morphologic features of DCE have been proposed by Rosenkrantz et al. for TZ prostate cancer and PI-RADSv2. A malignant pattern was described as “unencapsulated sheetlike or confluent” in contrast to “encapsulated swirled or popcorn-like enhancement.” Two readers included these patterns in PI-RADSv2 and found an upgrade in 2 of 6 or 4 of 7 TZ cancers ≥ Gleason 7, respectively. The reference standard was targeted biopsies [11].

PI-RADSv2.1 provides clarification for T2w and DWI criteria in TZ cancers. Additional morphologic characteristics of perfusion patterns have not yet been described in PI-RADSv2.1. Morphologic criteria may overcome the difficulty of differentiation of BPH nodules and TZ cancer.

Perfusion patterns in the prostate have to be correlated to histopathologic findings. A pattern of cancer can be described by increased cellular density with decreased luminal volume and extracellular space. These changes, as well as neoangiogenesis in the cancer lesions, anticipate a difference in perfusion pattern compared to BPH, which should be more complex beyond the time–signal curve. The time–signal curve reflects the *early* perfusion. In PI-RADSv2.1, this yields the distinction between DCE positive or negative. The PI-RADS definition also includes morphologic aspects and consideration of the surrounding tissue by the description of *focal* lesions that “correspond to suspicious finding onT2w and/or DWI” [4].

We propose a perfusion pattern that expands and specifies morphologic evaluation in a fast visual assessment of the DCE sequence in an early perfusion image alone.

The proposed features are asymmetry, the signal strength compared to the surrounding tissue, and the internal homogeneity of a focal lesion meaning a loss of the benign glandular pattern.

**Fig. 7:**
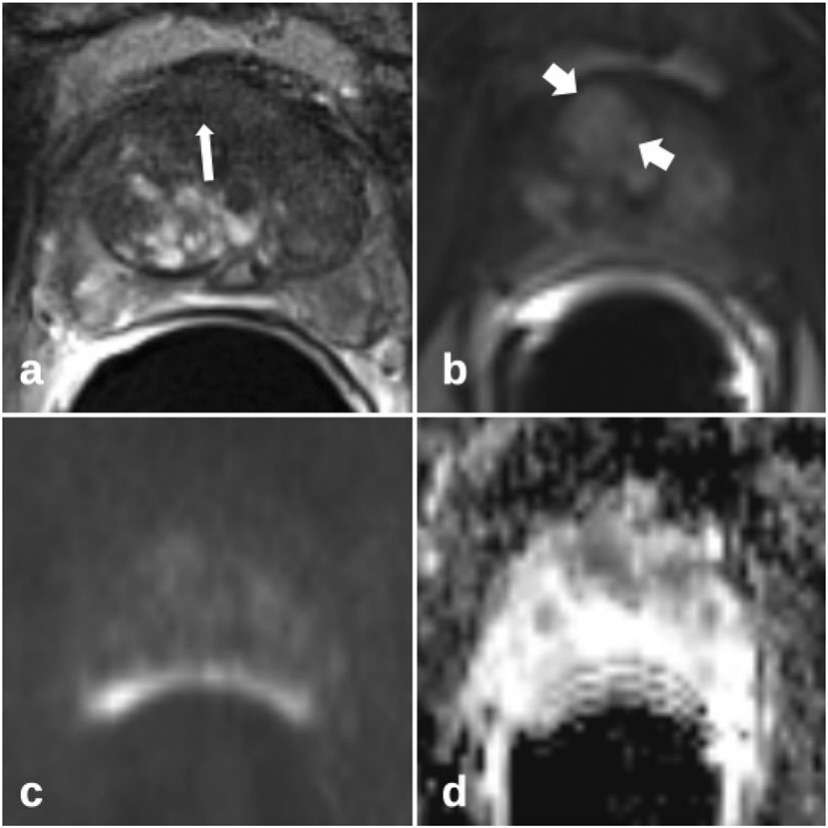
Homogenous perfusion pattern of transition zone prostate carcinoma (short arrows in **b**) with additional asymmetry improves lesion detection compared to T2-weighted and diffusion-weighted imaging alone; patient of 70 years, PSA 29 ng/ml, prostate volume 41 ml, prostate carcinoma G3 Gleason 4 + 4; **a** T2-weighted axial imaging with marked biopsy trace (long arrow); **b** T1w early-phase dynamic perfusion; **c** diffusion-weighted imaging with b = 1400; **d** apparent diffusion coefficient map

Asymmetry has already been described as a key feature in the differential diagnosis of cancer in the anterior fibromuscular stroma and the central zone [8]. The anterior fibromuscular stroma is usually hypovascular, and the central zone shows less early enhancement than the TZ. Therefore, it seems appropriate to apply the feature asymmetry to characteristic perfusion patterns to detect cancer that may extend into the anterior fibromuscular stroma (Fig. 2). BPH is characterized by nodules with a varying ratio of glandular to stromal tissue. Particularly, mixed and stromal BPH nodules with low T2w signal can mimic TZ cancer. Stromal nodules may also show early enhancement. PI-RADSv2.1 describes homogeneity with or without encapsulation as features for the differentiation between atypical nodules and suspect lesions. Stromal nodules are better defined, with an encapsulated appearance. It seems also appropriate to apply the feature homogeneity versus internal heterogeneity to characteristic perfusion patterns, similar to the typical T2w pattern of BPH. Figure 2 shows a typical TZ cancer with loss of heterogeneity compared to an unspecific perfusion pattern of BPH in Fig. 1. Marked homogeneity can also be described in Fig. 7.

Perfusion patterns were not evaluated separately for clinically significant TZ cancer, as the sample size was moderate. A significant portion of the positive diagnostic results were attributed to Gleason 3 + 3 scores (32 out of 77, 42%). In addition, the clinical outcome in transition zone cancer is better than in the peripheral zone. The lesion size may play a greater role in the clinical care and surveillance of Gleason 3+3 cancer. All the more, a better understanding of imaging criteria in the transition zone could improve active surveillance. The median lesion size in this study was 14 mm, and the minimum lesion size described was 5 mm. It is proposed that the typical perfusion pattern regarding heterogeneity or homogeneity needs a minimum size. Smaller nodules may be overlooked.

The results show that the evaluation of homogeneity in single early-enhancement images is not inferior compared to the conventional DCE parameter of PI-RADSv2.1 (AUC 0.84 versus 0.83).

This result is remarkable, as the perfusion pattern was evaluated in a single early perfusion phase image, whereas the DCE along PI-RADS was rated in the presence of all multiparametric image data.

Asymmetry and signal intensity as a single perfusion pattern failed to determine between carcinoma and no carcinoma. Signal strength in perfusion images depends on the selection of the perfusion phase and the vascularization of the surrounding TZ tissue. In an early-phase perfusion image, the signal strength reflects an early enhancement, as proposed in PI-RADSv2.1. The highest AUC of the multivariate model implies that the best differentiation of cancer is ensured by including perfusion pattern, T2w, and DWI.

### Limitations

There are some limitations in this retrospective single-center study.

The number of cancer-positive cores was much less than the number of cancer-negative cores due to the retrospective character of this study.

There was no wholemount section histology available, but the in-bore MRI-guided biopsy may reflect a suitable reference, as we get a direct correlation between image characteristics and histology.

The performance of perfusion patterns in single early perfusion images depends on the selection of the optimal phase. This was based on a subjective assessment.

## Conclusion

We suggest perfusion patterns that significantly improve the diagnostic performance of PI-RADSv2.1 in TZ prostate cancer.

Asymmetry, signal strength, and internal homogeneity meaning a loss of the glandular perfusion pattern seem to be interesting features for a new assessment of perfusion images. Fast visual evaluation of single perfusion images can meet the requirement of PI-RADS to simplify interpretation. This encourages texture analysis of perfusion images.

Further evaluation of perfusion patterns for the prediction of clinically significant cancer is necessary. Our results may reignite the discussion on biparametric PI-RADSv2.1 without DCE in contrast to PI-RADSv2.1.
